# Barriers, Perceptions, and Predictors for Utilizing Health-Related Technology Among Individuals Receiving Radiation Oncology Care

**DOI:** 10.7759/cureus.106788

**Published:** 2026-04-10

**Authors:** Maria Serafini, Cory Heal, Hayden A Ansinelli, Christopher M Morrison, Quoc Anh Ho, Victor Gonzalez, Sun Yi, Charles C Hsu, Jared R Robbins

**Affiliations:** 1 Department of Radiation Oncology, University of Arizona College of Medicine - Tucson, Tucson, USA; 2 Department of Radiation Oncology, Arizona Center for Cancer Care, Phoenix, USA; 3 Department of Radiation Oncology, Arizona Oncology, Tucson, USA; 4 Department of Radiation Oncology, University of Arizona Cancer Center, Tucson, USA; 5 Department of Radiation Oncology, University of California Irvine (UCI) Health Chao Family Comprehensive Cancer Center, Fountain Valley, USA; 6 Department of Radiation Oncology, PeaceHealth St. Joseph Cancer Center, Bellingham, USA; 7 Department of Radiation Oncology, University of Kentucky Markey Cancer Center, Lexington, USA; 8 Department of Radiation Oncology, Duke Cancer Institute, Durham, USA

**Keywords:** barriers to care, cancer patients, doctor - patient communication, mobile health applications, mobile technology

## Abstract

Introduction: For many people, mobile applications are pivotal to their daily lives. With a constantly increasing number of applications, the utilization of mobile health (mHealth) has augmented various aspects of clinical care outside of traditional clinical appointments, such as patient communication and monitoring. Despite these advancements, widespread adoption of these applications by cancer patients remains limited. This study aims to evaluate radiation oncology patients’ access to technology resources and evaluate the impact of the COVID-19 pandemic on willingness to engage in mHealth.

Methods: A survey-based study was conducted among 318 adult radiation oncology clinic patients between 2019 and 2022. Univariate and multivariate logistic regression were utilized to identify factors associated with willingness to utilize mHealth.

Results: On multivariate analysis, factors that impacted patients’ willingness to utilize mHealth include age>65 (OR 0.32; p=0.001), new patient status (OR 2.15; p=0.020), annual income>50K (OR 2.16; p=0.032), and smartphone ownership (OR 4.07; p=0.000). The post-COVID cohort showed an increased willingness to utilize mHealth compared to the pre-COVID cohort (OR 1.91; p=0.016). Lastly, a Technology Barrier Score (TBScore) was introduced to identify and quantify barriers that were associated with the reluctance to widespread adoption of mHealth.

Conclusion: Age, income, patient status, and smartphone ownership impacted radiation oncology patients’ willingness to utilize mHealth to report and track symptoms. Significant barriers include technology literacy and time commitment. The TBScore can serve as a valuable tool in identifying patients who may require additional support in the utilization of mHealth.

## Introduction

Technology and mobile applications have transformed and continue to change our society. From games to social media to wellness, there are apps available for almost any category of interest. As of 2020, more than 300,000 apps were dedicated to health, with an astounding 200 new apps being released daily [1]. Of these, approximately 40% were dedicated to patient care and management [1]. Healthcare professionals have found mobile health (mHealth) beneficial in allowing for remote access to electronic medical record (EMR) systems, communicating with other providers, and monitoring patients outside of the clinic [1]. Furthermore, the utilization of mHealth has increased and shown significant benefits with the COVID-19 pandemic in helping with prevention, screening, and treatment [2].

Despite the increased utilization and prevalence of mHealth, there are few apps dedicated to oncology patients. As a field that requires multidisciplinary longitudinal care, mHealth could play a major role in helping this patient population across different stages of treatment. One study found that about half of their oncology patients were willing to use mHealth and that age and gender were the strongest predictive factors. Males and younger demographics were more likely to use mHealth than females or older patients [3]. Another study found that the mHealth apps currently available were variable in the patient data that was tracked and that many apps were only dedicated to a specific cancer type or a specific institution [4].

This study aims to evaluate radiation oncology patients’ access to technology resources, the factors influencing mHealth adoption, and their willingness to use these resources to report health information. In addition, we measure the impact of the COVID-19 pandemic on patients’ readiness to engage in mHealth and introduce the Technology Barrier Score (TBScore) as a novel metric to identify individuals who may require additional support to effectively utilize these technologies.

## Materials and methods

A single-institution, cross-sectional survey study was conducted at the University of Arizona Cancer Center among adult radiation oncology clinic patients between 2019 and 2022. This study was approved by the University of Arizona Institutional Review Board (IRB # 1909003806). The base survey was divided into three parts to assess patient demographics, technology usage, and willingness to use mHealth for symptom tracking (Appendix 1). 

Two patient cohorts were analyzed: pre-COVID (2019) and post-COVID (beginning in September 2021, 20 months after the first confirmed case of COVID in the U.S.). The post-COVID cohort included three additional survey questions that addressed the impact of the pandemic on technology adoption.

Patient reluctance was assessed as a binary outcome and defined as selecting “not at all” or “not likely” (as opposed to "somewhat likely," "very likely," or "extremely likely") in response to the survey question, “How willing are you to use a mobile application to record/report your cancer symptoms and/or pain during treatment?”

Descriptive statistics, chi-square tests for categorical variables (p<0.05), and logistic regression models (including univariate and multivariate models) were used to assess predictors of technology adoption. The multivariate model adjusted for age, income, education, and smartphone ownership.

Using patient responses to the survey, the TBScore was developed as a novel scoring system to quantify technological barriers. The TBScore study identified key demographic and technological factors associated with reluctance to use mobile symptom reporting. The TBScore was calculated by assigning 0-2 points to significant univariate predictors based on the magnitude of their odds ratios (ORs). Predictors with an OR <1.5 were assigned 0 points (age <65, income ≥$50,000, completed post-secondary education, ownership of a smartphone/tablet/computer, and being a new or current patient). Predictors with ORs between 1.5-3.9 were assigned 1 point (age 65-74, income <$50,000, ≤ high school diploma, lack of tablet or computer ownership, and being a follow-up patient). Predictors with an OR ≥4 were assigned 2 points (age ≥75 or lack of smartphone ownership). Scores ranged from 0 to 9, with 0-2 classified as low barrier, 3-4 as moderate barrier, and 5-9 as high barrier toward mobile technology adoption. The statistical program utilized for analysis was Stata version 15 (StataCorp LLC, College Station, TX). Data were collected and maintained in RedCap (Vanderbilt University, Nashville, TN, Hosted by: University of Arizona).

## Results

A total of 318 patients completed the surveys (pre-COVID cohort=144 patients; post-COVID cohort=174 patients). The median age of the cohort was 65, and most patients were male (55.0%), self-identified as Caucasian individuals (75.0%), held a bachelor's degree (or completed some college) (60.1%), and had an annual income of <$50,000 (52.0%) (Table 1). Out of all 318 patients, 74.7% reported they used a smartphone (pre-COVID cohort=66.7%; post-COVID cohort=81.3%; p=0.005), 89.6% reported they had internet access at home (pre-COVID cohort=87.5%; post-COVID cohort=91.4%; p=0.34), and 85.5% reported using a computer (pre-COVID cohort=79.2%; post-COVID cohort=90.8%; p=0.005). 

**Table 1:** Demographic characteristics by cohort Demographic information and technology information for all 318 radiation oncology patients, stratified by pre-COVID and post-COVID cohorts.

Characteristic	All Cohorts (N=318)	Pre-COVID (N=144)	Post-COVID (N=174)	p-value
Age (years)				0.105
<56 yrs	42.9%	41.6%	44.1%	
56–74 yrs	38.4%	43.8%	33.9%	
75+ yrs	18.7%	14.6%	22.0%	
Gender				<0.001
Male	55.0%	52.2%	59.9%	
Female	42.0%	47.8%	40.1%	
Race				0.01
Caucasian	75.0%	72.2%	80.2%	
Other Races (combined)	25.0%	27.8%	19.8%	
Education Level				0.58
High School Graduate/GED	20.8%	22.6%	19.3%	
Bachelor’s Degree/some college	60.1%	56.9%	62.6%	
Graduate Degree	19.2%	20.4%	18.1%	
Income				0.02
<50,000 (combined)	52.0%	62.7%	47.7%	
≥50,000	41.0%	31.4%	45.9%	
Not Reported	7.0%	6.0%	7.6%	
Technology Access				
Smartphone Use	74.7%	66.7%	81.3%	0.005
Internet Access at Home	89.6%	87.5%	91.4%	0.34
Computer Use	85.5%	79.2%	90.8%	0.005

In univariate analysis, factors that influenced reluctance to use mHealth were older age (reference <65 years; 65-74 years: OR=2.88, 95% CI (1.46, 5.67), p=0.002; ≥75 years: OR=6.55, 95% CI (2.94, 14.60), p<0.001), lower income (<$50,000: OR=2.40, 95% CI (1.29, 4.47), p=0.002), lower education level (≤high school diploma: OR=1.84, 95% CI (1.01, 3.37), p=0.05), and lack of device ownership. More specifically, not owning a smartphone (OR=4.78, 95% CI (2.70, 8.44), p<0.001), tablet (OR=3.62, 95% CI (2.09, 6.27), p<0.001), or computer (OR=3.42, 95% CI (1.77, 6.60), p<0.001) significantly reduced the likelihood of reporting symptoms via mHealth (Table 2). 

**Table 2:** Univariate analysis of factors influencing reluctance of mHealth utilization The reference groups are age <65 years, income ≥$50,000, education >high school diploma, and device ownership present.

Factors	OR	95% CI	p-value
Age 65-74	2.88	(1.46, 5.67)	p=0.002
Age ≥75	6.55	(2.94, 14.60)	p<0.001
Income <50,000	2.40	(1.29, 4.47)	p=0.002
Education ≤High School	1.84	(1.01, 3.37)	p=0.05
No Smartphone	4.78	(2.70, 8.44)	p<0.001
No Tablet	3.62	(2.09, 6.27)	p<0.001
No Computer	3.42	(1.77, 6.60)	p<0.001

In multivariate analysis (Table 3), factors that impacted patients’ willingness to report symptoms with mHealth were: age>65 (OR 0.32; 95% CI (0.17, 0.61), p=0.001), new/current patient status (OR 2.15; 95% CI (1.13, 4.09), p=0.020), annual income>$50,000 (OR 2.16; 95% CI (1.07, 4.35), p=0.032), and smartphone ownership (OR 4.07; 95% CI (2.10, 7.89), p=0.000). The barriers preventing utilization of mHealth included limited technology literacy (p=0.024) and time commitment (p=0.048). The pre-COVID cohort reported privacy as a barrier more frequently than the post-COVID cohort (OR 2.3 vs. OR 1.1), trending towards significance. The post-COVID cohort was more willing to utilize mobile applications for symptom reporting (pre-COVID cohort=69.1%; post-COVID cohort=81.1%, OR 1.91; p=0.016). This remained significant on multivariate analysis after adjusting for age, concern for privacy, tech literacy, and patient status (OR 1.88; p=0.026). 

**Table 3:** Multivariate analysis of mHealth adoption factors Multivariate analysis of factors influencing adoption of mHealth, with an increased OR indicating increased reluctance to adopt mHealth. The reference groups are age <65, follow-up patient, income <$50,000, and no smartphone ownership.

Factors	OR	95% CI	p-value
Age >65	0.32	(0.17, 0.61)	p=0.001
New/Current Patient	2.15	(1.13, 4.09)	p=0.020
Income >50,000	2.16	(1.07, 4.35)	p=0.032
Smartphone Ownership	4.07	(2.10, 7.89)	p=0.000

Compared to the pre-COVID cohort, post-COVID participants demonstrated significantly greater access to mobile and online health applications with greater smartphone use (66.7% vs 81.3%, p=0.005) and computer use (79.2% vs 90.8%, p=0.005). Post-COVID respondents were more likely to be male (p<0.001), Caucasian individuals (p=0.01), and have higher household income (p=0.02), with no differences in education level (p=0.58) or age distribution. These factors taken together likely contributed to less reluctance to use mobile applications post-COVID compared to pre-COVID (18.4% vs. 32.6% unlikely to use, p=0.006).

**Figure 1:**
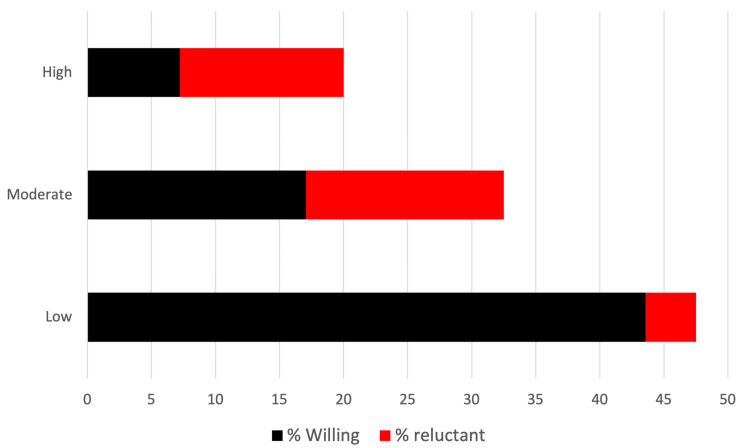
TBScore and reluctance to utilize mHealth Stacked bar chart depicting the distribution of patients by TBScore and the corresponding within-group reluctance to utilize mHealth. Each horizontal bar represents the proportion of the total patient cohort in each TBS group (low=47.5%, moderate=32.5%, high=20.0%). TBScore: Technology Barrier Score

A TBScore was assigned based on OR>4 (2 points), OR 1.5-3.9 (1 point), and OR<1.5 (0 points) and combined for a total of 0-9, where 0-2 was low reluctance, 3-4 was moderate reluctance, and 5-9 was high reluctance. There were 47.5% of patients in the low barrier group, 32.5% in the moderate barrier group, and 20.0% of patients in the high barrier group. Within these groups, 8.3% of low-barrier, 47.5% of moderate-barrier, and 63.9% of high-barrier patients were reluctant to report symptoms with a mobile device (p<0.05). These correspond to 3.94%, 15.44%, and 12.78% of the total cohort, respectively (Figure 1). Compared to patients in the low barrier group, patients in the moderate barrier group (OR: 3.35, p=0.002) and high barrier group (OR: 19.65; p<0.001) were significantly less willing to report symptoms with a mobile device. 

## Discussion

mHealth applications have grown rapidly in the last 10 years, with the increased significance of smartphones in daily life [5]. With approximately 800 oncology-related applications available, only 27% of them are intended for patient use, with only 18% of these intended for patient support [5]. This study aimed to evaluate the factors that influenced mHealth utilization among radiation oncology patients before and after the COVID-19 pandemic and to introduce the TBScore.

We found that various factors contributed to a reluctance to utilize mHealth by radiation oncology patients. These included older age, lower income, lower education level, lack of device ownership (specifically smartphones), and limited technology literacy. These findings are consistent with the extensively documented impact of social determinants on health outcomes and further support the “digital divide," where individuals with a lower socioeconomic status (SES) benefit less from mHealth compared to those of a higher SES [6]. Our findings are also supported by previous studies [7], where poor digital literacy and lack of internet access were some of the major contributing factors to the digital divide. 

Notably, the pre-COVID-19 cohort was more reluctant to utilize mHealth due to privacy concerns compared to the post-COVID-19 cohort, and smartphone usage increased in the post-COVID-19 cohort. These findings were consistent with the post-COVID cohort’s increased willingness to utilize mHealth for symptom-reporting. These changes before and after COVID may reflect shifting attitudes during the pandemic, when telehealth became essential for healthcare providers to deliver care. 

Limitations 

This study has several limitations. First, it is a cross-sectional study, which limits our ability to follow long-term trends for mHealth adoption. Second, the patient population was confined to radiation oncology patients at a single institution, which may affect generalizability both across geographical locations and to other oncologic disciplines. Third, a majority of the patients in this study were Caucasian patients and had a bachelor’s degree, potentially further limiting applicability to a more diverse patient population. Finally, because the TBScore was developed in a specific clinical setting, future work aims to externally validate this metric in other clinical environments and patient populations to ensure broader applicability. Once validated, the TBScore may serve as a practical tool for identifying patients who may benefit from additional support in adopting and using mHealth applications.

## Conclusions

In this study, we found several factors associated with an increased likelihood of adopting mHealth applications in a radiation oncology clinic setting. We introduced the novel TBScore as a predictive model for assessing patient reluctance/willingness to utilize mHealth applications. Patients in the moderate and high barrier groups were less willing to utilize mHealth compared to the low barrier group, suggesting that the TBScore could provide a valuable metric to identify patients who could more easily implement mHealth and those patients who may need further support. Ultimately, the widespread adoption and utilization of mHealth is multifactorial, and future longitudinal research should be conducted to investigate and develop targeted interventions to minimize digital health disparities.

## Figures and Tables

**Figure 2:**
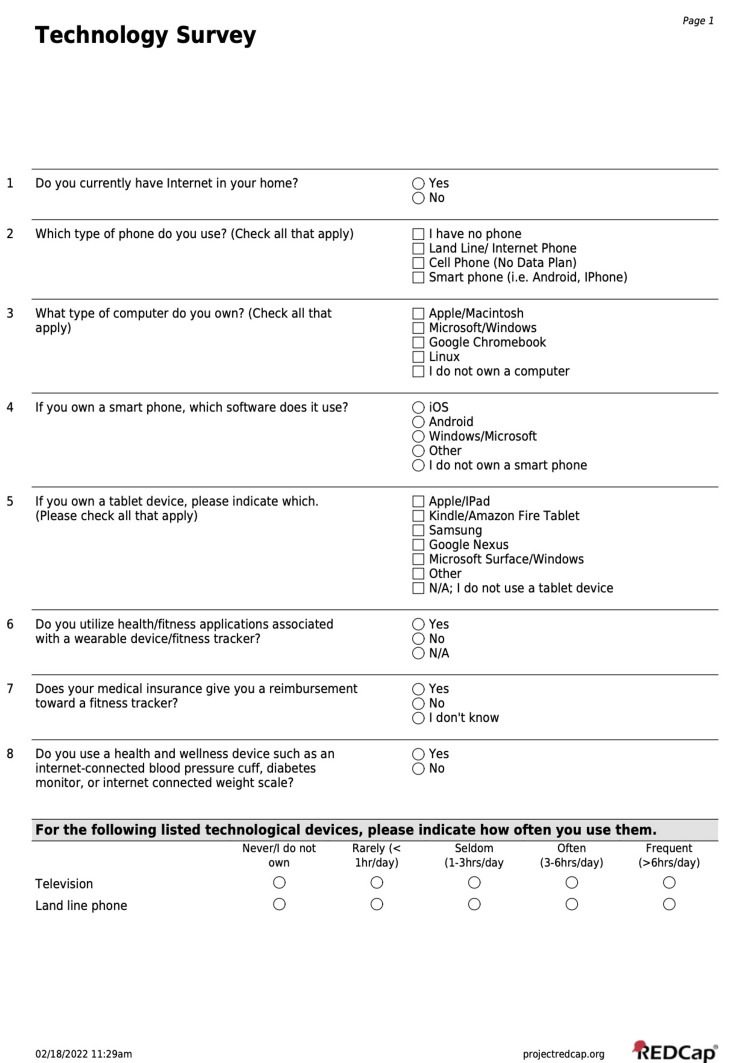
Survey (part 1)

**Figure 3:**
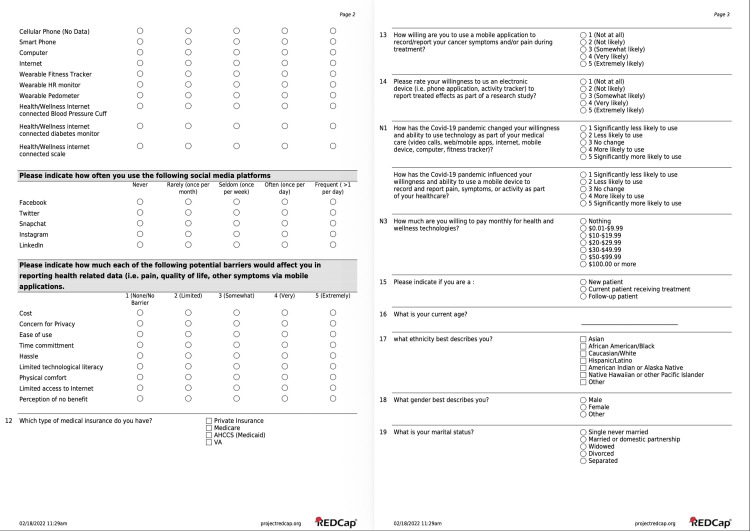
Survey (part 2)

**Figure 4:**
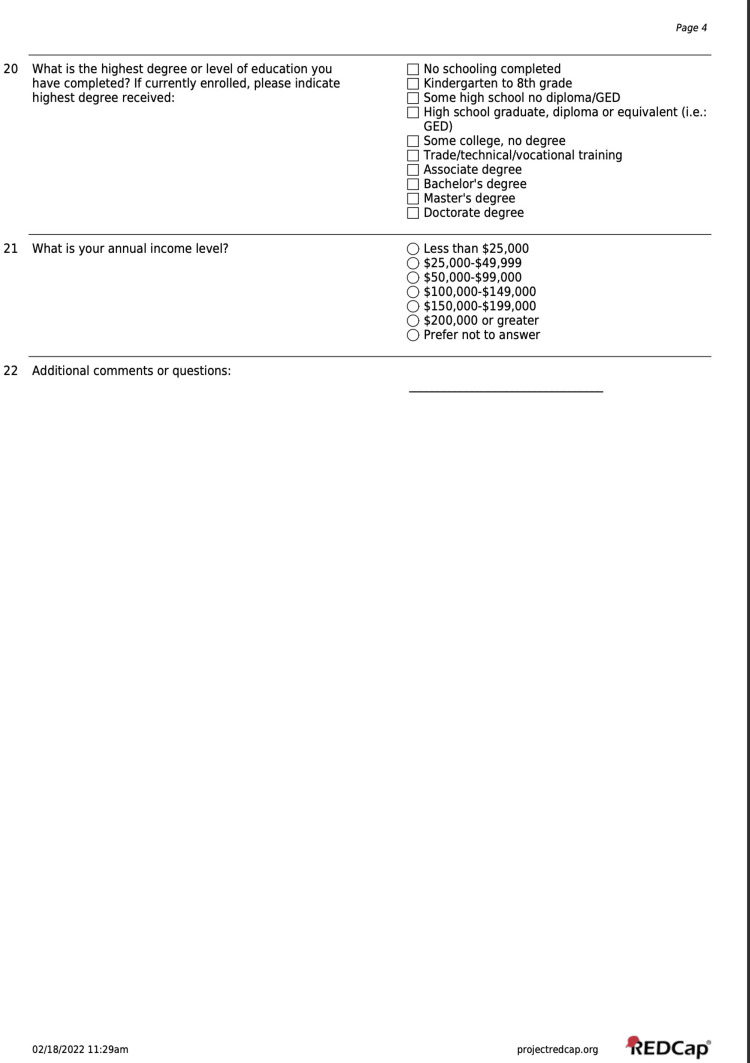
Survey (part 3)

## Appendices

Appendix 1
